# Type 2 Diabetes: Multiple Genes, Multiple Diseases

**DOI:** 10.1007/s11892-019-1169-7

**Published:** 2019-07-10

**Authors:** Miriam S. Udler

**Affiliations:** Massachusetts General Hospital Diabetes Center, 50 Staniford St, Suite 340, Boston, MA 02114 USA

**Keywords:** Type 2 diabetes, Subtypes, Genetics, Disease pathways, Polygenic risk score

## Abstract

**Purpose of Review:**

Type 2 diabetes (T2D), which accounts for the vast majority of diabetes cases, is essentially a diagnosis of exclusion in current clinical practice. Therefore, it is not surprising that T2D is heterogenous in terms of patients’ clinical presentation, disease course, and response to treatment. This review summarizes published attempts to improve diabetes subclassification, with a particular focus on the role of genetics.

**Recent Findings:**

A handful of diabetes subclassification schemas have been proposed using clinical data (patient characteristics and laboratory values), with some subgroups associated with distinct management trends or complication risks. However, phenotypically driven classifications suffer from dependencies on time of variable measurement and are not readily linked to disease mechanism. Germline genetic data, in contrast, are essentially unchanged over a person’s lifetime and rooted in mechanism. Clustering of T2D genetic loci has identified at least five groupings of loci representing mechanisms of disease that may aid in deconstructing heterogeneity of T2D, but further work is needed to determine clinical utility.

**Summary:**

Exciting progress in subclassification of diabetes has demonstrated initial steps in deconstructing disease heterogeneity. Incorporation of genetics into classification schemas will require additional research but has the potential to improve our understanding and management of T2D, both as a single disease and as a part of an integrated metabolic disease network.

## Introduction

In current clinical practice, when a patient develops elevated blood glucose indicative of diabetes, the diagnostic process of determining the diabetes “type” typically involves initially assessing for causes other than type 2 diabetes (T2D). For example, detection of autoantibodies may point to type 1 diabetes (T1D) or latent autoimmune diabetes in adults (LADA) or the presence of glucocorticoids on the medication list might suggest glucocorticoid-induced hyperglycemia. If a specific reason for hyperglycemia is not identified, a patient will generally then be considered to have T2D. Indeed, in practice, T2D is a diagnosis of exclusion, yet it currently is estimated to account for approximately 90% of all cases of diabetes [1]. It is not surprising, therefore, that T2D is a highly heterogenous condition with patients varying considerably in clinical presentation and response to treatment [2]. The heterogeneity observed among patients with T2D likely reflects variable contributions from diverse genetic and environmental factors [3], and ongoing efforts have aimed to utilize clinical and molecular data to develop a rational and reproducible categorization of diabetes. The goal of such subclassification is not only to refine patient diagnosis but also to better inform clinical management, specifically as it relates to prevention of diabetes complications. This review will summarize the diabetes-subtyping schemas that have been proposed as tools for deconstructing the heterogeneity of disease, with a particular focus on the role of genetics and its potential to shape our understanding and management of T2D, both as a single disease and as a part of an integrated metabolic disease network.

## Evidence for Type 2 Diabetes Constituting “Multiple Diseases”

The stereotypical phenotype of a patient with T2D is someone obese with evidence of insulin resistance; however, diabetologists know well that not all patients with T2D fit this mold. Likewise, not all patients with presumed T1D present with diabetic ketoacidosis (DKA) and have positive islet autoantibodies [1]. This recognition of disease heterogeneity has prompted several efforts to refine the classification of diabetes.

In 2003, Maldonado et al. presented the “AB” scheme, which was proposed to categorize patients presenting with DKA [4], and provided a useful construct for illustrating the diversity of these patients, who traditionally would have been assumed to have T1D. In 103 patients of various ethnic backgrounds who were admitted to the hospital with DKA, the authors assessed for presence of islet autoantibodies (A+/−) and evidence of beta-cell functional reserve (B+/−). They found that 50% of the patients were A−B+, 22% A−B−, 17% A+B−, and 11% A+B+ [4]. With only 17% of patients displaying antibody positivity and reduced beta-cell functional reserve (typical of T1D), the majority of these patients did not fit with the classic phenotypic picture associated with DKA. Furthermore, the substantial representation beyond both A−B+ (typical of T2D) and A+B− (typical of T1D) clearly demonstrated that patients with diabetes developing DKA did not fit neatly into well-established disease categories and that different pathophysiologies underlay their diabetes [5]. Of additional note, individuals in the B+ and B− groups also differed significantly by age of onset, glycemic control, and duration of insulin dependence, suggesting that recognition of subtype had clinical implications [4]. The utility in capturing beta-cell function in classifying diabetes “types” was also supported by a “beta-cell centric classification schema” later proposed by Schwartz et al., which conceptualized at least 11 pathways causing beta-cell dysfunction, each of which the authors envisioned could be targeted using a tailored treatment strategy [2].

Diversity among clinical phenotypes leading to development of diabetes was also evidenced in the work of Hulman et al. analyzing multi-point oral glucose tolerance tests (OGTT) in 5861 individuals without diabetes for whom longitudinal data was available. While typically only the fasting and two-hour time points of the OGTT are considered for diagnosing diabetes in non-pregnant adults, this analysis incorporated a third 30-min time point and fit latent class mixed-effects models across the three time points to identify four distinct glucose trajectory patterns [6]. Of particular interest was a subgroup (group 3) comprising 13% of individuals who had non-elevated 2-h glucose values, but elevated 30-min values; after up to 13 years of follow-up, individuals in group 3 were found to have a fourfold increased risk of developing diabetes and almost twofold all-cause mortality risk compared with individuals with similarly low 2-h glucose values, but who had non-elevated 30-min glucose readings (group 1). Compared with group 1, group 3 had similar insulin sensitivity, but reduced first-phase insulin response [6]. Further work will clarify how these subgroups, particularly group 3, relate to disruption of specific mechanistic pathways.

A large-scale data-driven approach to subclassify T2D was undertaken by Li et al. using electronic medical record data for 2551 individuals with T2D and 73 clinical features for topology-based patient-patient network generation [7]. Three distinct subgroups of T2D emerged, which appeared to have distinct additional disease risks and also unique genetic associations. Limiting the translatability of the findings, however, the study did not include replication of these subgroups in another dataset, and classification of a non-study patient into one of these three groups would not be straightforward. Additionally, it is unclear how to interpret the three groups in terms of underlying disease mechanism or relevance to patients in so far as actionability. Future work might also benefit from inclusion of ancestral background in the modeling approach to ensure that the specified subgroups do not simply represent categorization of patients by ancestry. Nevertheless, this work provided an exciting example of the potential of large-scale data and machine learning approaches to subtype complex disease.

Most recently, Ahlqvist et al. developed a new framework for characterizing adult onset diabetes based on six clinical metrics measured in Scandinavian individuals at the time of diabetes diagnosis: glutamic acid decarboxylase (GAD) antibody, age, body mass index (BMI), hemoglobin A1c, homeostatic model assessments of beta cell function (HOMA2-B), and insulin resistance (HOMA2-IR) [8••]. By applying *k*-means and hierarchical clustering algorithms, they identified five reproducible subgroups of patients: a severe autoimmune form (capturing T1D and LADA), a severe insulin-deficient form, severe insulin-resistant form, mild obesity-related form, and mild age-related form. The subgroups differed in terms of escalation of therapy and complications; for example, individuals in the severe autoimmune and severe insulin deficient clusters had the shortest times to sustained insulin use, and those in the severe insulin resistance cluster had the highest risk of developing chronic kidney disease. In a selected set of known T2D-associated genetic loci, at least one variant (rs7903146 in *TCF7L2*) had significantly different effects across the clusters, potentially supporting that the clusters are rooted in different biological disease processes [8••].

Applying the same data-driven approach as Ahlqvist et al. [8••], a subsequent study by Dennis et al. was performed using two large existing trial datasets of individuals with T2D randomized to metformin, sulfonylurea, or thiazolidinedione therapy [9]. The authors generated the same five clusters as previously observed in Ahlqvist et al. and found that these clusters differed in terms of glycemic progression, incidence of kidney disease, and glycemic response to medications. However, importantly, they noted that the observed differences between clusters could be better captured by other simple continuous features. For example, compared with analyses using the clusters, a model using only age at diagnosis similarly explained glycemic progression, and baseline-estimated glomerular filtration rate was a better predictor of time to chronic kidney disease. Similarly, a simple model incorporating sex, age at diagnosis, baseline BMI, and baseline HbA1c outperformed the clustering approach with regard to predicting treatment response. The authors therefore argue that “precision medicine in type 2 diabetes is likely to have the most clinical utility if it is based on an approach of using specific phenotypic measures to predict specific outcomes, rather than assigning patients to subgroups” [9].While this approach of using specific phenotypic measures for targeted clinical queries has pragmatic appeal, there may be benefits still to recognizing subcategories of disease, such as elucidation of underlying pathophysiology and development of novel targeted treatments.

## Can the “Multiple Genes” Contributing to T2D Aid Subclassification?

Identification of subtypes of disease or clinical predictors rooted in mechanistic processes would lend themselves naturally to the promise of precision medicine: the notion that when a person's disease is not only well-characterized clinically, but also has a well-understood etiological basis, we can provide optimal, individualized management. T2D has a strong genetic basis, with heritability estimates ranging from 30 to 70% [10–12]. These heritability estimates capture genetic risk as well as shared familial, prenatal, and postnatal environmental exposures. Like other complex diseases, T2D is polygenic with thousands of germline genetic loci estimated to contribute to disease, including more than 200 that have been identified to date [13••]. Large-scale studies have helped shape our understanding of the genetic architecture of T2D, suggesting that common genetic variation is responsible for a significant proportion of disease risk and that causal variants at each locus are often non-coding, implicating a regulatory role [14••, 15••]. Given the non-coding nature of these genetic variants, it has been challenging to connect a given locus to specific regulatory elements, relevant gene(s), and tissue(s) [13••, 15••]; thus, translation of the hundreds of established T2D genetic loci into improved understanding of disease pathophysiology and clinical utility has been slow, leaving the potential of genomic medicine in diabetes currently unfulfilled. However, we are now in a time of unprecedented scale of genetic studies, access to large cohort and biobanks linking phenotype to genotype, and emerging technologies to interrogate genomics with high-throughput assays.

Nevertheless, a critical question to consider is whether genetics is relevant to T2D sub-classification. At this point in time, broadly across all complex diseases, the role of germline genetics (genetic variation that a person is born with and is present in essentially all cells of the body) in subclassification remains mostly speculative with few examples reaching patient care. It may be noted that genetics has found abundant clinical utility for complex disease in the realm of cancer, ushering highly effective targeted therapy; however, the gains achieved there have been largely using somatic genetic sequencing (capturing genetic variation that has occurred in specific cells during a person’s lifetime) for molecular characterization of tumors to guide management [16]. Thus, given the limited precedent for clinical use of germline genetics for subtyping complex disease, it is worth reflecting on the lines of evidence supporting why genetics is not only relevant to T2D subclassification but also offers benefits beyond those seen with solely phenotype-based approaches.

First, the utility of genetics in T2D subclassification is supported by the existence of monogenic diseases that are frequently misdiagnosed as T2D. Indeed, dozens of genes have been implicated in monogenic diabetes. These conditions present clinically either with diabetes being the predominant disease feature, as is seen with forms of maturity onset diabetes of the young (MODY) and neonatal diabetes, or with diabetes existing as part of a syndromic presentation, as with mitochondrial diabetes and Wolfram syndrome. The genotype-phenotype relationships for several monogenic diabetes conditions were recently reviewed in guidelines published by the International Society for Pediatric and Adolescent Diabetes (ISPAD) [17••]. Monogenic diabetes collectively accounts for at least 0.4% of all diabetes cases, with MODY being the most common type [17••, 18]. It has been estimated that close to 80% of individuals with MODY remain undiagnosed [19]; many are living with a misdiagnosis of T2D, since the age of onset and clinical features of patients with MODY can overlap with T2D. Of course, the single pathogenic variants conferring large disease risk seen in monogenic diabetes differ considerably from the common genetic variation associated with small incremental risk of T2D; however, the fact that patients with MODY are frequently misdiagnosed with T2D highlights that employing genetics to improve detection of MODY (and other forms of monogenic diabetes) would reduce the heterogeneity of T2D (and T1D) attributable to misdiagnosed monogenic disease.

Second, and related to the first point, is that even within categories of phenotypically defined disease subgroups, there may exist genetic heterogeneity that is clinically important. There are numerous examples across medicine of so-called phenocopies, diseases with indistinguishable phenotypic characteristics, but with distinct genetic causes (e.g., multiple endocrine neoplasia (MEN) types 1 and 4). Within diabetes, MODY provides an illustrative example: patients who are clinically similar in terms of gross phenotypic and biochemical features (i.e., normal to low BMI, diabetes onset before age 30, preserved beta-cell function, and without evidence of autoimmunity) may have monogenic diabetes caused by several different genes, including most commonly *GCK*, *HNF1A*, and *HNF4A*. While in expert hands, patients with different genetic forms of MODY may be distinguishable based on careful, deep phenotyping, a genetic diagnosis provides objective evidence of distinct disease entities that can be phenotypically similar. Furthermore, in the case of MODY, we are fortunate to have knowledge that the implicated genetic alteration can guide management (e.g., patients with *GCK* MODY are generally safe without diabetes treatment and those with *HNF1A/HNF4A* can often be transitioned from insulin to oral therapy with sulfonylureas) [18]. While we are concerned with polygenic risk in T2D (rather than monogenic risk seen in MODY), clinically relevant genetic heterogeneity may also exist within T2D disease subtypes that are clinically indistinguishable. In these situations, it is possible that our current inability to recognize heterogeneity within clinically similar appearing individuals is due in part to relevant biomarkers being unknown or unavailable, and genetics offers an agnostic as well as relatively holistic diagnostic approach.

Third, genetics may guide disease management. In contrast to clinically defined subtype, a genetically defined subtype of disease lends itself more readily to potential mechanism-focused management strategies. Again, it is important to note that single genetic perturbations associated with monogenic disease will typically impose greater downstream consequences and impact on disease risk than those associated with T2D (which have modest effect sizes, generally OR < 1.2). However, it is possible that there will be individuals in whom composite polygenic risk impacts one or more specific disease pathways profoundly, and thus, such pathways could be useful for targeting management [20].

Finally, in contrast to many clinical and laboratory features, germline genetic markers do not change throughout a lifetime and are unaffected by treatments or disease course. Thus, a test assessing genetic classification could be applied at any time during disease course, including years after initial diagnosis and medication initiation or even potentially prior to diagnosis such that a preventative strategy could be employed.

## How “Multiple Genes” Inform Understanding of T2D Disease Pathways

What have we learned about T2D pathophysiology from the era of large-scale genetic association studies? To date, important windows into disease mechanisms have been elucidated from either (1) select T2D loci containing genes already implicated in diabetes pathophysiology, including *PPARG*, *HNF1A*, *HNF4A*, *KCNJ11/ABCC8*, and *GCKR*, or (2) select loci that have undergone elaborate follow-up fine-scale mapping and functional work, including *SLC30A8*, *SLC16A11*, and *TM6SF2* [15••, 21–27].

In particular, the evolving story of the *TM6SF2* association with T2D provides an elegant example of genetics enabling discovery of a novel T2D disease mechanism. The *CILP2* locus containing multiple genes, including *TM6SF2*, was initially associated with T2D in a meta-analysis of T2D genome-wide association studies (GWAS) published in 2012 [27]. Subsequently, exome chip analysis containing protein-coding variants across the genome identified an amino acid–altering variant in *TM6SF2* p.Glu167Lys as significantly associated with T2D, and two variants resulting in amino acid substitutions in this gene (p.Glu167Lys and p.Leu156Pro) were driving the locus association signal [15••]. The same variant *TM6SF2* p.Glu167Lys was significantly associated with non-alcoholic fatty liver disease (NAFLD) in an independent exome-chip analysis [25]. The shared T2D- and NAFLD-increasing allele of this variant was also associated with higher circulating levels of alanine transaminase, a marker of liver injury, and with lower levels of triglycerides [25]. Functional experiments knocking down *Tm6sf2* in mice [25] as well as *TM6SF2* in human hepatoma cell lines [26] both supported that reduced gene expression increased liver triglyceride content and decreased secretion of triglyceride-rich lipoproteins from liver tissue, thus implicating *TM6SF2* in liver fat metabolism. These exciting findings provided a novel disease pathway of primary liver tissue origin leading to increased risk of both T2D and NAFLD, demonstrating the power of genetics to uncover disease mechanism.

While a growing number of causal genes have been confidently connected to GWAS loci, the majority of T2D loci remain unmapped [13••]. As an alternative strategy to connect GWAS loci to mechanistic pathways, recent studies, including work from our lab, have leveraged multiple variant-trait associations to generate groups of related genetic variants and subsequently infer disease pathways [15••, 28–30]. As mentioned previously, a major challenge in connecting GWAS loci to relevant pathways is that the loci include dozens of variants highly correlated with one another, most of which are non-coding. Thus, despite advances in fine-scale mapping [13••, 15••, 31], for most T2D loci, we currently do not know which genetic variants, regulatory elements, genes, or tissues are functionally relevant. Multi-trait cluster analysis offers an opportunity to bypass this missing information and connect variants directly to pathways via the pattern of trait associations. In this approach, each T2D locus is represented by a variant associated with T2D at genome-wide significance, and the diabetes-risk-increasing allele is interrogated for its effect on multiple different traits. A clustering approach can then be applied to group variants together which share similar patterns of associations across multiple traits. The two most recent clustering efforts [15••, 29••] have found that applying a “soft” clustering approach, where variants can belong to one or more clusters, rather than a “hard” clustering approach [28, 30], which requires that each variant only belong to one cluster, produced more readily interpretable results. The “soft” clustering approach appears to be well-suited for modeling complex disease biology, since it allows a given locus to impact one or more genes, which in turn may alter one or more disease pathways.

**Fig. 1:**
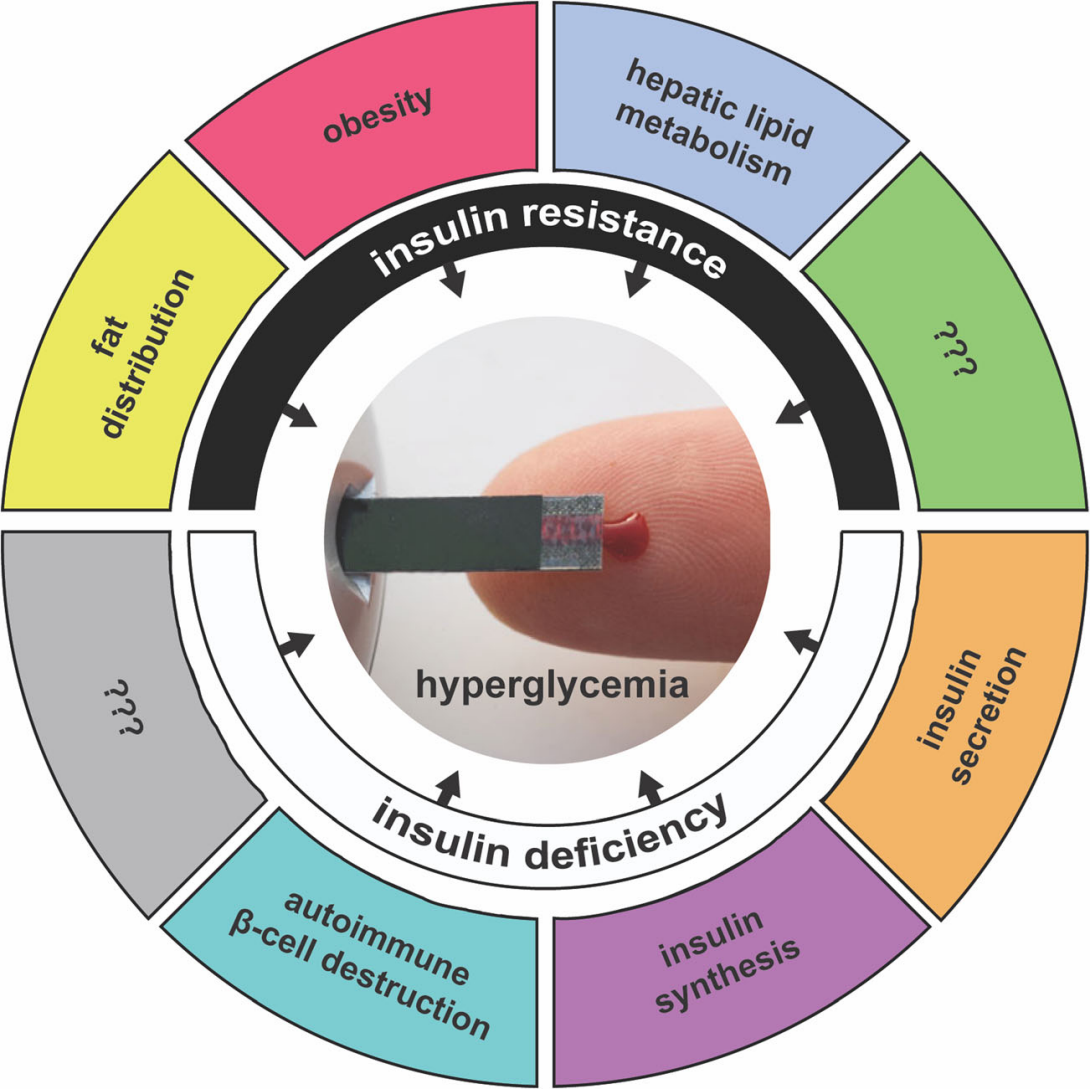
Based on the “Hallmarks of Cancer” presented in a landmark cancer biology paper by Hanahan and Weinberg [32], the different disease pathways contributing to diabetes are presented as the “Hallmarks of Diabetes.” Pathways are separated into mechanisms leading either to insulin deficiency or insulin resistance; insulin-independent mechanisms are not included here. The question marks indicate that additional disease pathways are yet to be identified

In Udler et al., clustering of 94 T2D variants and 47 metabolic traits using the soft clustering approach Bayesian non-negative matrix factorization (bNMF) produced five groupings of genetic loci (Fig. 1), each with distinct tissue-specific enhancer and promoter enrichment based on analysis of epigenomic data from 28 cell types [29••]. Two clusters contained variant-trait associations indicative of reduced beta-cell function, differing from each other by high vs. low proinsulin levels, suspected to represent defective insulin processing vs. defective insulin synthesis, respectively. The three other clusters of loci represent mechanisms of insulin resistance: obesity-mediated (high BMI and waist circumference), “lipodystrophy-like” fat distribution (low BMI, adiponectin, and HDL-cholesterol, and high triglycerides), and disrupted liver lipid metabolism (low-serum triglycerides). In line with the prior discussion of functional work supporting the role of *TM6SF2* in liver fat metabolism, this locus was most strongly weighted in the final liver lipid metabolism cluster. The clusters were enriched for active regulatory elements in tissues which were consistent with suspected pathways; for example, the defective insulin processing cluster was most strongly enriched for regulatory elements in pancreatic islet cells compared with the other 27 cell types (*P* < 0.001), and the disrupted liver lipid metabolism cluster was significantly enriched for elements in liver tissue (*P* < 0.001). The epigenomic data thus provided additional support that these genetically informed pathways represent distinct disease mechanisms [29••]. Additionally, the loci implicated in each cluster were differentially associated with other metabolic diseases: the defective insulin processing loci T2D-increasing alleles were most strongly associated with stroke risk and the lipodystrophy loci T2D-increasing alleles were most associated with increased systolic and diastolic blood pressure [29••].

Using another “soft” clustering approach called *C*-means, Mahajan et al. similarly analyzed GWAS data for a set of 94 T2D association signals partly overlapping with those used in Udler et al. [29••] and 10 T2D-related quantitative traits, identifying six variant clusters (shown in Supplementary Fig. 6b of [15••]). Five of the clusters broadly mapped to the same clusters from Udler et al. described above. Thus, reassuringly, two independent approaches of clustering T2D variant-trait associations resulted in largely similar findings, suggesting robustness in these genetically driven disease pathways.

In considering the role of genetics in elucidating disease mechanisms, a conceptual model proposed for cancer biology may be useful for diabetes biology. A landmark cancer biology paper by Hanahan and Weinberg describing the pivotal deregulated pathways underlying cancer development offers a framework to deconstruct the intricacies of this complex disease [32]. These functional pathways, termed the “Hallmarks of Cancer,” can perhaps inspire us to conceptualize the “Hallmarks of Diabetes” (Fig. 1), and consider how defining key diabetes pathways may likewise eventually guide disease classification and management. In the case of cancer, perturbations of these pathways (typically via somatic genetic alteration, potentially on a germline risk background) induce tumor development and sustained growth. Different cancers may display deregulation within specific functional pathways, highlighting disease etiology and guiding treatment selection. For example, tumors displaying deregulated apoptotic pathways are often responsive to drugs that induce cancer cell apoptosis [33]; tumors with defective DNA damage repair mechanisms may respond well to immune-directed therapies [34] or drugs that exploit genomic instability [35]; and cancers with abnormal signaling pathways can respond to kinase inhibitors [36]. Therefore, identification of key functional pathways within such a framework can reduce disease complexity, improve understanding of disease mechanism, and inform the development and clinical use of targeted therapies. By all means, cancer and diabetes disease biology have important differences, and the discussion presented here of diabetes biology is restricted to germline variation; however, cancer serves as a powerful example of how a holistic approach to defining pathway deregulation can be integrated with our understanding of genomic variation to transform the treatment of disease. As we continue to identify the pathways, or hallmarks, of diabetes, we can start to ask whether individuals develop disease predominantly through one or multiple pathways and whether these pathways can be targeted for treatment. Moreover, if in fact most individuals develop diabetes via deregulation of multiple pathways, as is suspected [20], a “poly-pill” combination therapy that targets multiple pathways could be beneficial.

**Fig. 2:**
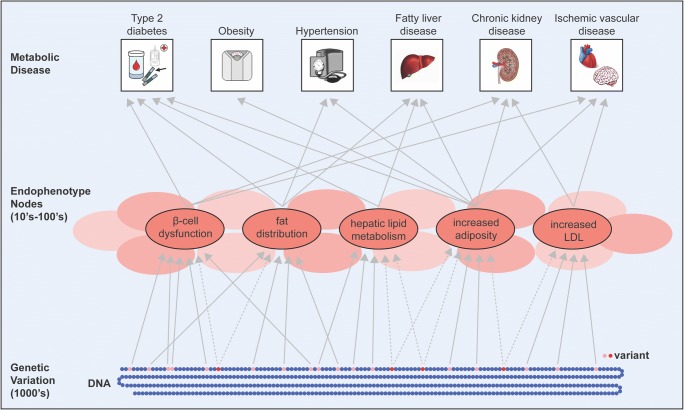
A future holistic vision for genetic variation contributing to metabolic conditions via disease processes, termed endophenotypes. Genetic variants shown at the bottom of the figure within DNA colored in red contribute to multiple endophenotypes whereas those in pink contribute to only one. It is hypothesized that across multiple metabolic diseases, thousands of distinct genetic signals contribute to dozens of endophenotypes. The endophenotypes represent targets for therapy and importantly targeting a given endophenotype may affect one or more metabolic disease conditions

As underlying disease mechanisms become elucidated, our conceptualization of metabolic disease will expand. T2D is, in a sense, an artificial construct based on a threshold chosen against the continuum of hyperglycemia. Likewise, other metabolic diseases, such as obesity and hypertension, are based on chosen thresholds for continuous traits (BMI and blood pressure measurements). The cluster-defined pathways leading to increased T2D risk also appear to impact risk of other metabolic conditions, raising the notion that shared disease processes or “endophenotypes” may underlie metabolic diseases (Fig. 2). As our knowledge of genetic variation impacting metabolic diseases continues to grow, clustering and other approaches will continue to refine our understanding of these critical endophenotypes, such as dysregulated insulin processing or unfavorable fat distribution. Appreciating the molecular basis for shared risk among metabolic diseases might help improve disease screening among unaffected individuals and also screening and/or preventative steps to reduce complications among those with T2D.

## Efforts to Use Genetics to Subtype T2D’s “Many Diseases”

Identification of mechanistic disease pathways causing T2D will no doubt be useful for improved understanding of pathophysiology and drug development. Beyond these benefits, a natural next question to ask is whether genetically defined clusters of loci can identify subtypes of diabetes. Genotype information could be used to identify individuals carrying many alleles within a given cluster, potentially representing multiple “hits” along a pathway. In this scenario, diabetes in these individuals would be driven predominantly by one or few pathways, and knowledge of the underlying disease pathway(s) could guide management.

At this point in time, such a genetic cluster-based approach to diabetes classification is not ready for application in the clinic. Our group has shown, however, that genetically defined subgroups of T2D can identify individuals with differing phenotypic traits, thereby serving as an initial step in deconstructing the heterogeneity of T2D [29••]. In Udler et al., pathway-specific genetic risk scores (GRS’s) for the five genetic clusters were generated for 17,365 individuals with T2D, combined across four cohorts, to determine whether individuals with the top 10% GRS uniquely for each cluster would have clinical differences. The cut-off of top 10% was arbitrarily chosen, and further work is needed to set more clinically relevant thresholds; however, differences between subgroups were observed using this 10% (“extreme”) threshold. Indeed, those patients with T2D with extreme GRS for the two insulin deficiency clusters had lower C-peptide levels than all other individuals with T2D (*P* < 0.01, combined); those with extreme obesity cluster GRS had significantly increased BMI, percent body fat, hip circumference, and waist circumference (*P* values < 0.05); those with extreme lipodystrophy cluster GRS had significantly decreased high-density lipoprotein cholesterol, percent body fat, and BMI (*P* values < 0.01); and those with extreme liver lipid metabolism cluster GRS had significantly decreased serum triglyceride levels (*P* = 0.01). Thus, individuals with T2D and a GRS uniquely at the top 10% of one cluster had representative trait characteristics collectively distinguishing them from all other individuals with T2D. Further work is underway to determine whether those at the highest percentile of a cluster respond differentially to any medications or are at differential risk for complications of diabetes.

Clinically relevant genetic subtyping for T2D might also involve utilizing a GRS developed for predicting T1D risk. In its most updated form, the 67-SNP T1D GRS was highly discriminative for identifying individuals with T1D (area under the receiver operator characteristics curve (AUC) of 0.92) [37]. Additionally, a T1D GRS may have utility in identifying individuals with later age onset T1D who are misdiagnosed as T2D [38] as well as predicting escalation to insulin therapy in patients with presumed T2D with GAD Ab positivity [39]. In the latter study, a 30-SNP T1D GRS was calculated in 8608 individuals diagnosed with T2D after 35 years of age and treated without insulin for at least 6 months following diagnosis. The T1D GRS predicted progression to insulin use at five years, but only in GAD-positive participants: probability of insulin use (95% CI): 47.9% for high T1D GRS (35.0%, 62.78%) vs. 27.6% for medium T1D GRS (20.5%, 36.5%) vs. 17.6% for low-risk T1D GRS (11.2%, 27.2%); *P* = 0.001 [39]. Interestingly, there was no association with insulin use at 5 years in GAD-negative individuals, which comprised the majority of study participants (96.7%). One can imagine that eventually, a more intricate model including T2D pathway-specific GRS as well as T1D pathway-specific GRS may be useful for improved modeling of diabetes subgroups.

## Conclusion

Exciting progress has been made in exploring approaches to “slice up” the large, approximately 90%, T2D portion of the diabetes subtype pie. The clinically defined subgroups proposed by Ahlqvist et al. [8••] will no doubt undergo deeper physiological and genetic characterization in the coming months, potentially leading to increased appreciation of the underlying mechanistic processes for each cluster. Additionally, with increased awareness of monogenic diabetes and access to genetic testing, the small slice of misdiagnosed monogenic diabetes cases will be chipped away from the T2D segment of the pie. It is likely that pathway-specific genetic GRSs will be further developed with clinically relevant thresholds determined, such that these “hallmarks of diabetes” can be utilized to further chip away at the T2D segment and guide management.

Challenges will remain. For example, placing a new individual into clinically derived clusters, such as those developed by Ahlqvist et al. [8••], is not straightforward in practice, particularly if distributions of the various clinical factors differ across populations (e.g., distributions of BMI or HbA1c may differ in patients with diabetes in Scandinavia compared with those in the USA). Applying the method to populations of different ancestries and development of online calculators will facilitate clinical translation. Pathway-specific genetic risk scores, likewise, have been developed based on data from individuals of European ancestry, and availability of GWAS summary statistics for analyses in diverse ancestral populations will be necessary for GRSs to be developed for individuals of non-European ancestry.

Finally, perhaps new approaches will be developed that combine clinical and genetic characteristics into a single model, ideally also incorporating environmental factors such as dietary and lifestyle habits. While inclusion of genetics into classification schemas will require additional research, it has the potential to improve our understanding and management of T2D, both as a single disease and as a part of an integrated metabolic disease network.
